# Evaluation of the cobas^®^ GT hepatitis C virus genotyping assay in G1-6 viruses including low viral loads and LiPA failures

**DOI:** 10.1371/journal.pone.0194396

**Published:** 2018-03-22

**Authors:** Benjamin Némoz, Léa Roger, Vincent Leroy, Jean-Dominique Poveda, Patrice Morand, Sylvie Larrat

**Affiliations:** 1 Laboratory of Virology, Institute for Biology and Pathology, Grenoble Alpes University Hospital, Grenoble, France; 2 Department of Hepatology, Grenoble Alpes University Hospital, Grenoble, France; 3 Cerba Laboratories, Cerba HealthCare, Saint-Ouen-l’Aumône, France; 4 Institut of Structural Biology (IBS) –Mixed Research Unit 5075 (CEA-CNRS-UGA), Grenoble, Grenoble, France; Kaohsiung Medical University Chung Ho Memorial Hospital, TAIWAN

## Abstract

Direct-acting antiviral (DAA) drug performances depend on the viral genotype. So international recommendations give typing of the virus a prerequisite for treatment choice and patient management. Commercially available HCV genotyping kits are scarce and this analysis is often in-house using tedious PCRs and Sanger sequencing, leading to a lack of standardization. A newly commercialized HCV genotyping assay based on real-time PCR has been developed by Roche Diagnostics (Mannheim, Germany). We compared this new assay with our in-house PCRs -sequencing technique on 101 regular samples and 81 LiPA failures or low viral load samples. No genotype or 1a/1b subtype mismatch was observed. Two samples were misidentified at the subtype level without clinical impact. Three genotype 1b and two genotype 1a samples with low viral load could not be subtyped. Nevertheless, 13 (13%) samples from the regular panel and 35 (43%) from the more difficult-to-type panels failed to give results on first pass with the Roche kit. Failures were mostly associated with genotype 3 subtype a, with genotype 4 subtype non-a, or with viral loads <200 IU/mL (p = 0.0061). The workflow allowed a non-specialized technician to obtain results in less than 4 hours whereas 2 to 3 days and experienced staff were required with the in-house assay. In conclusion, the Roche cobas^®^ HCV GT kit is easy and rapid to use and provides reliable results. The high rate of uninterpretable results particularly for low viral load samples and less frequent genotypes, and the absence of subtyping for non-genotype 1 could require sending complex samples to a specialized laboratory.

## Introduction

In 2015 the World Health Organisation estimated that about 71 million people are living with the hepatitis C virus [1]. Every year, about 700,000 people die of hepatitis C-related pathologies including hepatocellular carcinoma (HCC), cirrhosis and liver failure [2]. Recently, the availability of highly-active direct-acting antivirals (DAA) targeting non-structural viral proteins raised the hope of a rapid eradication of HCV. However, the asymptomatic nature of hepatitis C infection is a major problem for management of the disease and targeting the right persons with the right treatments is crucial in order to achieve a sustained virologic response (SVR) at a patient level. Recent recommendations stated that both the genotype and the subtype of the virus are critical for drug selection among those available. Thus, accurate genotyping now plays a key role in the management of HCV patients [3].

Commercially available kits for HCV genotyping are scarce. The most commonly used assays are the VERSANT HCV Genotype 2.0 assay (LiPA, Siemens Healthcare, Germany) that is based on a reverse hybridization line probe assay [4], and the RealTime HCV Genotype II kit (Abbott, Illinois, USA) that is based on fluorescent-labeled oligonucleotide probes [5]. In addition to these a few other kits are available for HCV genotyping [6]. The VERSANT HCV Genotype 2.0 assay requires specific equipment (Auto-LiPA Instrument, blot and scan) and presents some typing failures for genotype 1 subtypes [7] or undetermined results, especially for samples coming from non-European regions [8]. Moreover using 5’UTR and Core regions this assay cannot correctly identify the recombinant form 2k/1b [9]. The Abbott assay has been found to misclassify genotype 1 subtypes in 1–5.4% of samples [7,10]. Consequently, many laboratories still use in-house polymerase chain reaction (PCR) sequencing assays. There is a need for reliable commercially available assays so as to enhance standardization of HCV genotyping. The use of equipment already present in the laboratory for HCV viral load quantification, as for the Abbott assay, is an advantage. Roche Diagnostics has recently developed a new kit for HCV genotyping that allows the genotyping of genotypes 1 to 6 and the determination of subtypes 1a and 1b. It runs on the cobas 4800 platform that is already used for measuring HCV viral loads [11,12]. This method is based on real-time PCR for the amplification and uses specific fluorescent probes for the detection of the HCV genome.

The objective of our study was to evaluate the performances and workflow of the cobas^®^ HCV GT kit from Roche Diagnostics (Mannheim, Germany) in the context of a teaching hospital laboratory, compared with our in-house assay. We therefore evaluated this recently launched method on samples from both routinely managed patients and a selected panel of more complicated cases with sequenced viruses of less frequent genotypes.

## Materials and methods

### 1) Samples

A total of 182 samples from chronically infected HCV patients were retrospectively genotyped using the HCV GT Roche assay. Among them, 101 corresponded to an HCV typing analysis performed for patients followed in the Hepatology care unit of Grenoble Alpes University hospital between February 2014 and April 2016, and for whom more than 650 μL of plasma remained. The viral loads of the samples from this panel were always superior to 4 log10 IU/mL with a mean viral load of 6.00+/-074 log10 IU/mL [13]. A further eighty-one samples were added representing two categories of more difficult to type samples. These corresponded either to samples with low viral load (< 3 log10 IU/mL, n = 42, mean viral load: 1.92+/-0.44 log10 IU/mL), or to samples collected nationwide (n = 39, viral load unknown) received for expertise in our specialized laboratory between February 2014 and April 2016 following the failure of previous typing using the LiPA VERSANT HCV Genotype 2.0 assay. The characteristics of the latter samples are given in Table 1. Samples were stored in the local biobank (DC2008-680) at -80°C. No patient was asked to give an additional blood sample.

**Table 1:** Distribution of Hepatitis C virus (HCV) genotypes in studied samples (n = 182).

	HCV genotype and subtype	Number of samples (n = 101)	HCV Mean Viral load (log10 IU/mL+/-sd)
**Regular samples**	1a	35	6.04+/-0.79
	1b	30	6.21+/-0.62
	2c	2	5.59+/-1.25
	3a	11	5.78+/-0.84
	4a	7	6.26+/-0.24
	4d	12	5.48+/-0.66
	4 non-a non-d	3	6.23+/-0.05
	6e	1	6.90
**Low viral load samples**	1a	13	2.03+/-0.46
	1b	16	1.92+/-0.39
	1e	1	1.3
	3	6	1.83+/-0.45
	4	6	2.00+/-0.46
**LiPA failures**	1d	2	/
	1g	3	/
	1i	1	/
	2i	9	6.04+/-0.43
	2b	1	/
	2c	1	/
	2k	1	/
	RF 2k/1b	1	/
	3h	2	/
	4f	7	/
	4b	2	/
	4k	1	6.02
	4n	1	/
	4o	1	/
	4q	1	5.01
	4r	2	/
	5a	3	/

### 2) Ethics statement

The collection of biological samples (authorized collection DC2008-680) was approved in December 2008 and reconfirmed in December 2013 by the bioethics committee of the French Ministry of Higher Education and Research. All clinical investigations were conducted according to the principles expressed in the Declaration of Helsinki and the study was approved by the local ethics committee (Comité de Protection des Personnes, Sud-Est V IRB0006705). All patients gave signed informed consent for the use of data and samples for research at the beginning of their hospital stay, as part of the institutional procedures. Authors BN and SL had access to full identification of samples, including full names of patients. Samples were then de-identified prior to any analysis. Other authors did not have any access to identification of samples.

### 3) Sequencing method

All samples had been previously genotyped using our in-house sequencing assay considered here as the reference assay. Extraction of RNA from plasma samples was performed using the NucliSENS^®^ EasyMAG system from BioMérieux (Marcy l’Etoile, France). A total volume of 1 mL of plasma was used for the extraction and nucleic acids were eluted in 50 μL. Extracted RNA was stored at -80°C until analysis and re-frozen after RT-PCR and sequencing for eventual re-analysis.

First, nucleic acid amplification was performed in the NS3 region of the HCV genome as described by Besse *et al* [14]. Amplification was checked on 1% agarose gels with the GelRed intercalant. When no amplification was obtained, the NS5B region was amplified as described by Sandres-Sauné *et al* [15]. Finally, and only for samples from the difficult to type panels, an amplification of the Core region was performed as described in Pham *et al* [16]. The sequencing reaction was then performed using the Beckman Dye Terminator Cycle Sequencing (DTCS) kit (Beckman Coulter, California, USA), and sequence detection was made by capillary electrophoresis in a Beckman Coulter capillary electrophoresis CEQ 8000 system.

Newly obtained sequences were first blasted against the whole nucleotide NCBI database [17]. The Max Planck Institute Geno2Pheno [HCV] tool was then used to confirm the genotype [18]. A final step of phylogenetic analysis was performed using the MAFFT alignment tool [19] from the Japanese Computational Biology Research Consortium. The phylogenic tree was built using reference sequences described in Murphy *et al* [20].

Our sequencing assay is regularly assessed by the Quality Control for Molecular Diagnostics (QCMD) scheme (catalogue code: QAV034117). The results from the evaluations received while this study was conducted are highly supportive of our assay. Results form 2016 and 2017 evaluations reported a score of zero (highest grade) for every sample tested.

### 4) Roche cobas^®^ HCV GT

The method evaluated here is the kit (Roche cobas^®^ HCV GT) specifically designed to be used on the Roche cobas^®^ 4800 automated platform. RT-PCR was performed on a Z480 thermocycler from the same automated platform [12]. The cobas 4800 system is currently CE marked and FDA approved but the cobas 4800 HCV GT assay is only CE marked and not yet FDA approved. Detection of HCV samples was made by the same thermocycler, and the signal was processed through a proprietary interface [21].

The Roche HCV GT assay is designed to differentiate all genotypes from 1 to 6 and also to determine 1a, 1b and 1 non-a non-b subtypes. No typing of subgroups within genotypes 2, 3, 4 or 6 is possible and genotypes 7 are not recognized.

The sample volume required for analysis is 400 μL of plasma with 250 μL of dead volume for automated pipetting. Each sample is distributed between three reaction wells for RT-PCR.

The PCR is performed using the kit’s proprietary designed primers. Amplification of genotypes 2, 3 and 6 relies on the 5’UTR region. For genotypes 1, 4 and 5, the Core region of the genome is amplified and detected. Finally, discrimination between genotypes 1a and 1b is done by amplification of the RNA-dependent-RNA-polymerase (NS5B) gene.

Detection of both the HCV genome and its genotype is achieved using fluorescent hydrolysis probes. Internal controls are incorporated in the kit and allow technical validation. A positive control containing extracts from genotypes 1, 4 and 6 allow monitoring of the three RT-PCR reactions. A negative control is also included in the kit. Failure to amplify and detect HCV genotypes is highlighted by the software with three different outcomes: samples considered as ‘Invalid’ are amplified and detected but invalid internal controls prevent the results from being released. Samples indicated as ‘Indeterminate’ are amplified and detected but the genotype cannot be accurately determined. Samples indicated as ‘Failed’ were not correctly extracted or did not perform well in the pre-analytical phase and must be re-tested.

## Results

Samples included in this study were distributed as follows: Genotypes 1 made up 55.5% (n = 101) of the samples. These included subtype 1a (n = 48, 26.4%), subtype 1b (n = 46, 25.3%) and non-a non-b subtype (n = 7, 3.8%). The remaining 81 samples were distributed among the other genotypes: genotype 2 (n = 14, 7.7%), 1 recombinant form 2k/1b, genotype 3 (n = 19, 10.4%), genotype 4 (n = 43, 23.6%) and genotypes 5 and 6 (n = 4, 2.2%). The full distribution of genotypes in the three panels tested is listed in Table 1. Altogether, results could be obtained for 74.7% of the samples in a first-pass and for 77.5% after re-testing failed samples. The cases of samples that failed to give results in the first run and gave results when retested could be explained by either the presence of a clot in the sample or by an insufficient volume of plasma in the analysis tube.

Among the 101 samples in the regular genotyping panel, 88 (87.1%) were accurately identified by the Roche kit (S1 Table). Results from both techniques are shown in Table 2. When a result was obtained with the Roche kit, there was no mismatch in identification at either the genotype level or at the subtype level. The remaining 13 samples failed to be amplified (“failed” n = 9, 8.9%) or to be correctly classified (“indeterminate” n = 4, 4.0%). Eight of these samples could be re-tested thanks to a sufficient volume of remaining plasma. Among these 8 samples, 5 gave accurate results on second test and 3 had repeated amplification failure. The invalid results were due to a failed internal control that could be due to an inhibition of the PCR reaction in at least one of the 3 PCR steps but why some results were obtained when retested remains unclear [21]. Most of the samples which failed were from genotypes 1a (4/35, 11%), 3a (3/11, 27%) or 4d (3/12, 25%). Mean viral load among these failed samples was not significantly different from the rest of the panel (6.5 log10 IU/mL, p = 0.801). Determination of genotype 6 was explored with only one sample of subtype 6e. This sample was not correctly detected by the Roche kit, the system giving an ‘indeterminate’ result for the sample even though the viral load was elevated (6.9 log10 IU/mL).

**Table 2:** Correspondence between results obtained with both techniques for the regular sample panel (n = 101).

		Roche HCV GT genotyping results									Total
		1	1a	1b	2	3	4	5	6	NI ^b^	
Sequencing technique results	1a		31							4	35
	1b			29						1	30
	2c				2					0	2
	3a					8				3	11
	4a						6			1	7
	4d						9			3	12
	4k						1			0	1
	4n						1			0	1
	4r						1			0	1
	6e								0	1	1

NI: Not interpretable (Failed, invalid or indeterminate)

Among the LiPA failed samples, only 61.5% (n = 24) of the tested samples were correctly genotyped and subtyped by the Roche cobas 4800 kit (Table 3). Four samples could be retested and results were obtained for two of them with a correct genotype classification. Two samples gave different results with both techniques at the subtype level (S2 Table). The Roche technique found both to be genotype 1b. However, our in-house sequencing technique classified them as 1d. They were the only two cases for which a difference between the techniques was highlighted. One was checked by resequencing the sample on a different HCV genomic region thanks to the remaining sample volume confirming a 1d subtype. We tested only one recombinant genotype where the sample was determined as 2k/1b by our in-house sequencing technique. The kit identified it correctly as both genotypes 1b and 2. Among the 13 (33.3%) uninterpretable samples, 1 was invalid (1 genotype 4k at 6.02 log10 IU/mL), 7 were considered as indeterminate (4 genotypes 1 non-a non-b, 2 genotypes 3h and 1 genotype 4o) and 5 as failed (4 genotypes 4 non-a non-d and 1 genotype 2c).

**Table 3:** Correspondence between results obtained with both techniques for the LiPA failed samples (n = 39).

	Roche HCV GT genotyping results										Total
		1	1a	1b	2	3	4	5	6	NI	
Sequencing technique results	1g									3	3
	1d			2						0	2
	1i									1	1
	2i				9					0	9
	2b				0					1	1
	2c				1					0	1
	2k				1					0	1
	2k/1b			1	1					0	1
	3h					0				2	2
	4f						5			2	7
	4b						1			1	2
	4k						0			1	1
	4n						1			0	1
	4o						0			1	1
	4q						1			0	1
	4r						1			1	2
	5a							3		0	3

NI: Not interpretable (Failed, invalid or indeterminate)

In the whole study, a total of 7 samples were genotype 1 non-a non-b, but none of them was correctly identified by the Roche kit despite correct PCR amplification. Five of those samples gave no result (indeterminate), and the two remaining were the 1d misclassified samples.

**Fig 1:**
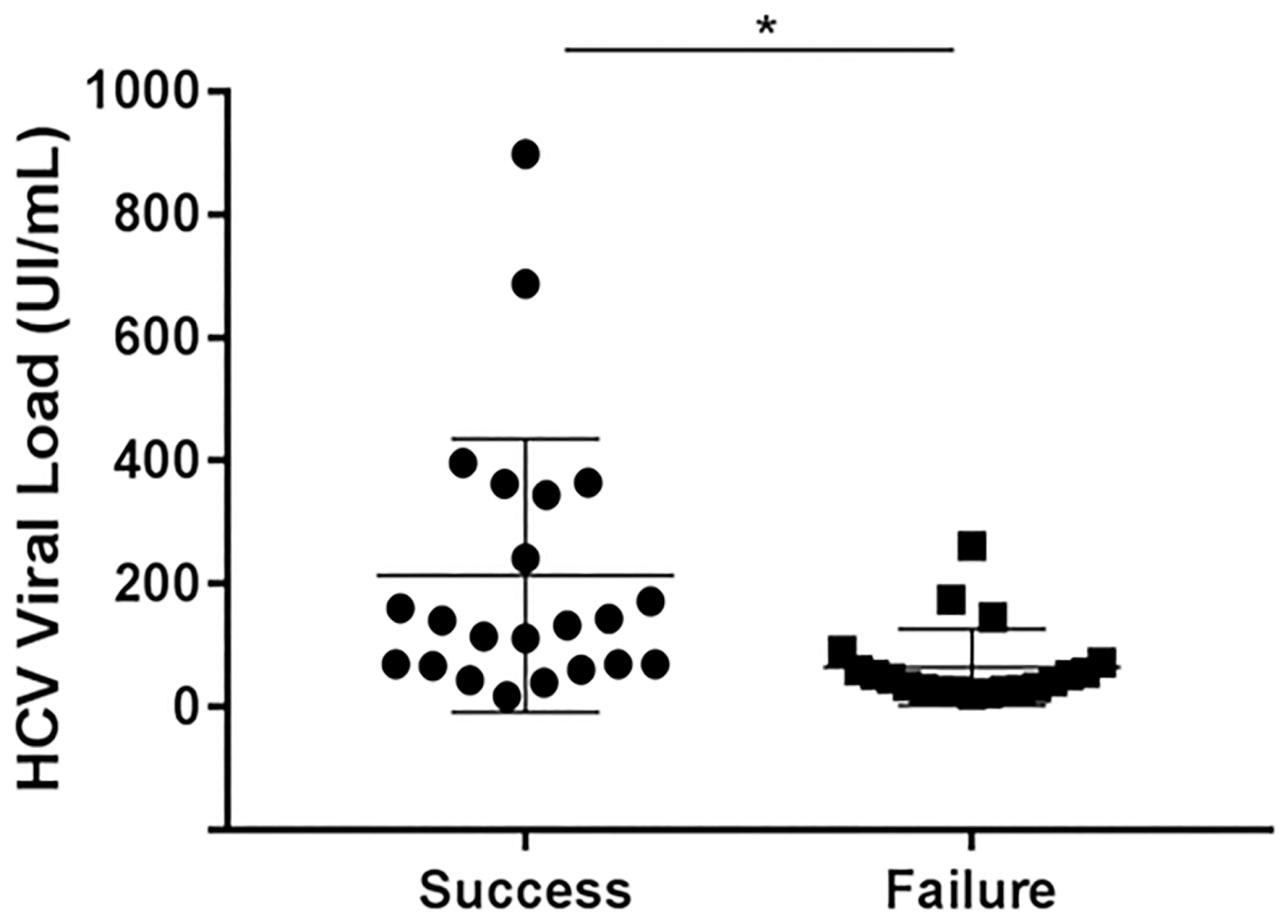
Genotyping results with Roche HCV genotyping kit on samples with low HCV viral load. (p = 0.006) Successes: filled circles; failures: filled squares; (p = 0.006).

For viral loads < 3 log10 IU/mL genotyping assay success was correlated with the viral load (p = 0.0061) (Fig 1) and failures (20/42) mostly occurred when it was < 2.3 log10 IU/mL [200 IU/mL] (S3 Table). Surprisingly, these missing results are essentially indeterminate and invalid thus indicating the correct amplification of the sample (Table 4).

**Table 4:** Correspondence between results obtained with both techniques for the low viral load samples (n = 42).

		Roche HCV GT genotyping results									Total
		1	1a	1b	2	3	4	5	6	NI	
Sequencing technique results	1a	2	4							7	13
	1b	3		8						5	16
	1e									1	1
	3a					3				3	6
	4a						2			1	3
	4d						0			2	2
	4g						0			1	1

NI: Not interpretable (Failed, invalid or indeterminate)

We also evaluated the kit in terms of its ease-of-use and integration into the workflow of our laboratory. The current organization of our in-house procedure leads to the extraction and reverse transcription phases being done on the first day. Then PCR amplification is performed on the second day with control of the amplification by gel electrophoresis. The sequencing reaction is usually done on day three and results are released on the fourth day after verification of chromatograms and analysis. Moreover, experienced staff are required. The Roche kit handles samples in a maximum runtime of three and a half hours. The operator does not need particular skills above those of a laboratory technician and their presence is only required at three different steps (loading of samples and reagents, transfer of extracted samples to the amplification and detection system, and validation of results), for a total of 30 minutes including technical validation. The release of results can be made 4 to 5 hours after the beginning of the run (pretreatment of samples not included), including biological validation.

## Discussion

Like the previous studies published on this assay, our study confirmed the good accuracy of the Roche HCV genotyping assay with no genotype or subtype 1a/1b mismatch when compared to our in-house PCR-sequencing HCV genotyping assay [12,22,23].

When results were available the overall agreement between methods was 98.53%. An exploitable result was available for 74.7% of samples (87.1% for the routine panel, 66.7% forLiPA failures and 52.4% for low viral load samples) in a first-pass analysis.

Also no strong conclusion can be drawn considering the relative small number of samples for each genotype, the kit developed by Roche Diagnostics was unable to discriminate between subtypes of genotypes 2 to 6 nor to accurately identify subtypes of genotype 1 that differ from 1a or 1b. Among the 101 genotype 1 samples tested in this study, 94 were accurately identified between 1a or 1b subtypes, two were inaccurately subtyped (1b/1d), and 5 gave indeterminate results.

This work also highlighted a quite high rate of uninterpretable results during the first pass (from 13% for regular samples to 52% for viral loads inferior to 1000 IU/mL). Re-testing of failed samples allowed the number of samples without results to be reduced but required a sufficient volume of remaining plasma. Stelzl *et al*. reported only 3.8% of indeterminate samples [12]. Our results on the routine panel exhibited the same number of indeterminate results (4%) but this number increased in low viral load and LiPA failure panels. Moreover we took into account the total number of samples for which no result was obtained. These uninterpretable results corresponded mainly to genotype 3 and 4 non-a samples.

The manufacturer’s instructions about viral loads set different lower limits of detection for each genotype. The range varies from 2.1 log10 IU/mL [125 IU/mL] for genotype 1a to 3 log10 IU/mL for genotype 5 (Table 5). We tested 42 samples below this threshold. Samples with viral load below the manufacturer’s limit failed in 47.6% (n = 20) of cases. Interestingly, 17 out of the 42 samples gave interpretable and accurate results. Nevertheless, most HCV genotyping will be performed before introduction of antiviral drugs, when viral loads are usually high.

**Table 5:** Characteristics of the commercially available HCV genotyping assays.

	cobas^®^ HCV GT	Siemens Versant HCV GT 2.0	Abbott RealTime HCV GT II
Technology	Real time PCR	Reverse hybridization technology	Real time PCR
Target regions	5’UTR, NS5B, Core	5’UTR, Core	5’UTR, NS5B
Sensitivity (LoD)	**Plasma samples:** GT 1a,2,3,4,6: 125 IU/mL GT 1b: 250 IU/mL GT 5: 1000 IU/mL **Serum samples:** GT 1a,1b,3,4,6: 125 IU/mL GT 2: 50 IU/mL GT 5: 500 IU/mL	2,106 IU/mL	500 IU/mL
Genotype coverage	1–6, 1a/b	genotypes 1–6 and subtypes 1a vs. 1b, and subtypes 6 (c-I)	1–6, 1a/b
Sample processing volume	400 μL	500–1000 μL	500 μL

Other failures were mostly due to less common genotypes (1d, 1e, 1g, 1i, 2b, 2i, 2k, 3h, 4b, 4d, 4f, 4g, 4k, 4n, 4o, 4q, 4r) which had already failed when using the LiPA VERSANT HCV Genotype 2.0 assay. We have already described the failure of this last technique to type non-European samples due to the non-specificity of the probes used in 5’UTR and Core regions [8]. Despite the addition of amplification in the NS5B region, these samples also exhibited a very high rate of failure with the Roche assay (13/39) most likely due to mismatches with the primers or the probes used. For 5 samples, amplification failed, pointing to primers mismatches. This was mostly the case with genotype 4 samples, probably due to the variability of the Core region used for amplification. Hovwever, the other 8 samples failed to be detected, highlighting the limitations of the probes used. On the other hand, the conjunction of targets on the 5’end of the HCV genome (5'UTR and Core) and on the 3’end of the genome (NS5B) allows the Roche assay to detect 2k/1b recombinant forms.

Taken together our results are in line with the findings of other teams [12, 22, 23].

Unlike Nieto-Aponte *et al*, we did not observe any double-infection. Stelzl *et al* reported an overall agreement of 87.4% on a similar number of plasma samples [12]. Fernández-Caballero *et al* showed more successful results on a comparable number of samples tested, but they did not include rare subtypes [23]. Our results show more discrepancies between our in-house sequencing technique and the Roche kit, with up to 26.4% samples unidentified. This is mainly due to our use of the kit at the limits of recommended viral load, as well as the selection of rare genotypes.

Unlike PCR-sequencing assays, this new HCV genotyping kit is unable to recognize any new genotype, such as the recently-described genotypes 7a, 7b or 8a [24–26]. Embedded controls, which are the same than for viral load assay, will detect the viral RNA but no specific probe is included resulting in an uninterpretable result.

Due to its design, the Roche assay also does not differentiate genotype 1 subtypes other than 1a/1b. Hence, subtyping was not as precise as that available with the sequencing method. At present, the international recommendations do not differentiate anti-HCV treatments according to subtype with the exception of genotypes 1. In the near future, despite the arrival of theoretically pan-genotypic therapeutic combinations, some differences may persist such as the lower efficacy of sofosbuvir/velpatasvir/voxilaprevir for 8 weeks on genotype 1a or of the glecaprevir/pibrentasvir combination on previously treated genotype 3a [27–29]

It has also been shown in previous studies that different subtypes of genotype 4 viruses behave differently in terms of the virologic response patients can achieve. Treatment naïve patients with subtype 4d or 4r showed significantly more failures in virologic response than those with subtype 4a [30,31].

The latest editions of both the American [32] (AASLD—IDSA) and the European recommendations [3] (EASL), point out the necessity of looking for resistance associated mutants in the NS5A region of the HCV genome for the treatment of genotype 1a virus using elbasvir/grazoprevir. This will require the addition of another assay.

Our study highlighted a few weaknesses of the Roche assay, but the number of samples in each genotype or subtype was too low to draw definitive conclusions as to assess the failure to type one particular subtype and more studies focusing on each of the variants will be needed. Genotype 3a samples were scarce despite being one of the most prevalent genotypes found worldwide, the chosen viral panels being strongly influenced by local epidemiology. Therefore, more data need to be gathered on this genotype to give a full overview of the Roche technique. We did not include any double infection by two different HCV strains in the tested samples and this also should be tested in further studies.

With regard to the implementation of the Roche assay in a medical virology laboratory, we found staff training was rapid (1 day) and the instruments needed can also be used for HCV, HBV, and HIV viral load measurements. Unlike our current technique based on customized RT-PCR and Sanger sequencing for which operator time is high, the Roche assay was fast and not time-consuming. As results can be obtained in less than 4 hours, this ease-of-use gives it a distinct advantage over in-house PCR-sequencing assays that require experienced staff, as well as several days before obtaining a result. Of note, if genotyping failed with the Roche assay, the next step was to perform in-house sequencing. Therefore the total working hours became more than 5 days. Nevertheless, considering the costs of implementing this assay, while no complete economic study was performed, it is likely that reduced hands-on time by trained technical staff will lead to reduced costs as seen by Nieto-Aponte *et al*. [22].

In conclusion, the cobas 4800 HCV GT assay from Roche provides a rapid, easy-to-use and accurate solution to first line genotyping of HCV. PCR-sequencing assays performed by an expert center will still be required when the Roche assay fails or when precise data on subtype or NS5A resistance associated mutations are needed for therapeutic decision-making.

## Supporting information

S1 Table Raw data for the regular samples. This file includes sequencing results, results obtained with Roche assay on first and second pass and viral load for each sample.
(DOCX)

S2 Table Raw data for the LiPa failed samples. This file includes sequencing results, results obtained with Roche assay on first and second pass and viral load for each sample.
(DOCX)

S3 Table Raw data for the low viral load samples. This file includes sequencing results, results obtained with Roche assay on first and second pass and viral load for each sample.
(DOCX)

## References

[1] WHO. Global hepatitis report, 2017 [Internet]. 2017 apr. Report No.: ISBN: 978-92-4-156545-5. http://www.who.int/hepatitis/publications/global-hepatitis-report2017/en/

[2] Global, regional, and national age–sex specific all-cause and cause-specific mortality for 240 causes of death, 1990–2013: a systematic analysis for the Global Burden of Disease Study 2013. The Lancet. 2015;385(9963):117–171. 10.1016/S0140-6736(14)61682-2 PMC4340604 25530442

[3] European Association for Study of Liver. EASL Recommendations on Treatment of Hepatitis C 2015. J Hepatol. 2015;63(1):199–236. doi: 10.1016/j.jhep.2015.03.025 25911336 10.1016/j.jhep.2015.03.025

[4] Verbeeck J, Stanley MJ, Shieh J, Celis L, Huyck E, Wollants E, et al Evaluation of Versant Hepatitis C Virus Genotype Assay (LiPA) 2.0. J Clin Microbiol. 2008;46(6):1901–1906. doi: 10.1128/JCM.02390-07 18400913 10.1128/JCM.02390-07 PMC2446848

[5] Michelin BDA, Muller Z, Stelzl E, Marth E, Kessler HH. Evaluation of the Abbott RealTime HCV assay for quantitative detection of hepatitis C virus RNA. J Clin Virol. 2007;38(2):96–100. doi: 10.1016/j.jcv.2006.11.007 17185031 10.1016/j.jcv.2006.11.007

[6] Germer JJ, Majewski DW, Rosser M, Thompson A, Mitchell PS, Smith TF, et al Evaluation of the TRUGENE HCV 5’NC Genotyping Kit with the New GeneLibrarian Module 3.1.2 for Genotyping of Hepatitis C Virus from Clinical Specimens. J Clin Microbiol. 2003;41(10):4855–4857. doi: 10.1128/JCM.41.10.4855-4857.2003 14532242 10.1128/JCM.41.10.4855-4857.2003 PMC254363

[7] Chueca N, Rivadulla I, Lovatti R, Reina G, Blanco A, Fernandez-Caballero JA, et al Using NS5B Sequencing for Hepatitis C Virus Genotyping Reveals Discordances with Commercial Platforms. PLoS One. 2016;11(4):e0153754 doi: 10.1371/journal.pone.0153754 27097040 10.1371/journal.pone.0153754 PMC4838212

[8] Larrat S, Poveda J-D, Coudret C, Fusillier K, Magnat N, Signori-Schmuck A, et al Sequencing Assays for Failed Genotyping with the Versant Hepatitis C Virus Genotype Assay (LiPA), Version 2.0. J Clin Microbiol. 2013;51(9):2815–2821. doi: 10.1128/JCM.00586-13 23616453 10.1128/JCM.00586-13 PMC3754683

[9] Ramière C, Tremeaux P, Caporossi A, Trabaud MA, Lebossé F, Bailly F, et al Recent evidence of underestimated circulation of hepatitis C virus intergenotypic recombinant strain RF2k/1b in the Rhône-Alpes region, France, January to August 2014: implications for antiviral treatment. Eurosurveillance [Internet]. 2014;19(43). Available from: http://www.eurosurveillance.org/content/10.2807/1560-7917.ES2014.19.43.20944 10.2807/1560-7917.es2014.19.43.20944 25375898

[10] Chevaliez S, Bouvier-Alias M, Brillet R, Pawlotsky J-M. Hepatitis C Virus (HCV) Genotype 1 Subtype Identification in New HCV Drug Development and Future Clinical Practice. PLoS One. 2009;4(12):e8209 doi: 10.1371/journal.pone.0008209 19997618 10.1371/journal.pone.0008209 PMC2785465

[11] Vermehren J, Stelzl E, Maasoumy B, Michel-Treil V, Berkowski C, Marins EG, et al Multicenter comparison study of both analytical and clinical performance across 4 Roche HCV RNA assays utilizing different platforms. J Clin Microbiol. 2017;55(4):1131–1139. doi: 10.1128/JCM.02193-16 28122870 10.1128/JCM.02193-16 PMC5377840

[12] Stelzl E, Appel HM, Mehta R, Marins EG, Berg J, Paar C, et al Evaluation of the new cobas^®^ HCV genotyping test based on real-time PCRs of three different HCV genome regions. Clin Chem Lab Med. 2017;55(4):517–521. doi: 10.1515/cclm-2016-0620 27740913 10.1515/cclm-2016-0620

[13] Kessler HH, Cobb BR, Wedemeyer H, Maasoumy B, Michel-Treil V, Ceccherini-Nelli L, et al Evaluation of the COBAS^®^ AmpliPrep/COBAS^®^ TaqMan^®^ HCV Test, v2.0 and comparison to assays used in routine clinical practice in an international multicenter clinical trial: The ExPECT study. J Clin Virol. 2015;67:67–72. doi: 10.1016/j.jcv.2015.03.023 25959162 10.1016/j.jcv.2015.03.023

[14] Besse B, Coste-Burel M, Bourgeois N, Feray C, Imbert-Marcille BM, André-Garnier E. Genotyping and resistance profile of hepatitis C (HCV) genotypes 1–6 by sequencing the NS3 protease region using a single optimized sensitive method. J Virol Methods. 2012;185(1):94–100. doi: 10.1016/j.jviromet.2012.06.011 22728274 10.1016/j.jviromet.2012.06.011

[15] Sandres-Sauné K, Deny P, Pasquier C, Thibaut V, Duverlie G, Izopet J. Determining hepatitis C genotype by analyzing the sequence of the NS5b region. J Virol Methods. 2003;109(2):187–193. 12711062 10.1016/s0166-0934(03)00070-3

[16] Pham DA, Leuangwutiwong P, Jittmittraphap A, Luplertlop N, Bach HK, Akkarathamrongsin S, et al High prevalence of Hepatitis C virus genotype 6 in Vietnam. Asian Pac J Allergy Immunol. 2009;27(2–3):153–160. 19839502

[17] Zhang Z, Schwartz S, Wagner L, Miller W. A Greedy Algorithm for Aligning DNA Sequences. J Comput Biol. feb 2000;7(1–2):203–214. 10.1089/10665270050081478 10890397

[18] Kalaghatgi P, Sikorski AM, Knops E, Rupp D, Sierra S, Heger E, et al Geno2pheno[HCV]–A Web-based Interpretation System to Support Hepatitis C Treatment Decisions in the Era of Direct-Acting Antiviral Agents. PLoS One. 2016;11(5):e0155869 doi: 10.1371/journal.pone.0155869 27196673 10.1371/journal.pone.0155869 PMC4873220

[19] Kuraku S, Zmasek CM, Nishimura O, Katoh K. aLeaves facilitates on-demand exploration of metazoan gene family trees on MAFFT sequence alignment server with enhanced interactivity. Nucleic Acids Res. 2013;41(W1):W22–8. 23677614 10.1093/nar/gkt389 PMC3692103

[20] Murphy DG, Willems B, Deschenes M, Hilzenrat N, Mousseau R, Sabbah S. Use of sequence analysis of the NS5B region for routine genotyping of Hepatitis C Virus with reference to C/E1 and 5’ untranslated region sequences. J Clin Microbiol. 2007;45(4):1102–1112. doi: 10.1128/JCM.02366-06 17287328 10.1128/JCM.02366-06 PMC1865836

[21] Roche Diagnostics. Package insert: cobas^®^ HCV GT [Internet]. Report No.: 07564414001-01FR. https://molecular.roche.com/assays/cobas-hcv-gt-for-use-on-the-cobas-4800-system/

[22] Nieto-Aponte L, Quer J, Ruiz-Ripa A, Tabernero D, Gonzalez C, Gregori J, et al Assessment of a novel automatic real-time PCR assay on the cobas 4800 analyzer as a screening platform for hepatitis C virus genotyping in clinical practice: comparison with massive sequencing. J Clin Microbiol. 2017;55(2):504–509. doi: 10.1128/JCM.01960-16 27927921 10.1128/JCM.01960-16 PMC5277520

[23] Fernández-Caballero JA, Alvarez M, Chueca N, Pérez AB, García F. The cobas^®^ HCV GT is a new tool that accurately identifies Hepatitis C virus genotypes for clinical practice. PLoS One. 14 4 2017;12(4): 3–7. e0175564. 10.1371/journal.pone.0175564 PMC5391928 28410425

[24] Salmona M, Caporossi A, Simmonds P, Thélu M-A, Fusillier K, Mercier-Delarue S, et al First next-generation sequencing full-genome characterization of a hepatitis C virus genotype 7 divergent subtype. Clin Microbiol Infect. 2016;22(11):947.e1–947.e8. 27515394 10.1016/j.cmi.2016.07.032

[25] Murphy DG, Sablon E, Chamberland J, Fournier E, Dandavino R, Tremblay CL. Hepatitis C Virus Genotype 7, a New Genotype Originating from Central Africa. J Clin Microbiol. 2015;53(3):967–972. doi: 10.1128/JCM.02831-14 25520447 10.1128/JCM.02831-14 PMC4390628

[26] Hedskog C. Identification of novel HCV genotype and subtypes in patients treated with sofosbuvir based regimens—C. AASLD, abstract 210. oct 2017; https://liverlearning.aasld.org/aasld/2017/thelivermeeting/201395/charlotte.hedskog.identification.of.novel.hcv.genotype.and.subtypes.in.html

[27] Kumada H, Watanabe T, Suzuki F, Ikeda K, Sato K, Toyoda H, et al Efficacy and safety of glecaprevir/pibrentasvir in HCV-infected Japanese patients with prior DAA experience, severe renal impairment, or genotype 3 infection. J Gastroenterol. 2017 doi: 10.1007/s00535-017-1396-0 29052790 10.1007/s00535-017-1396-0 PMC5866827

[28] Kwo PY, Poordad F, Asatryan A, Wang S, Wyles DL, Hassanein T, et al Glecaprevir and pibrentasvir yield high response rates in patients with HCV genotype 1–6 without cirrhosis. J Hepatol. 2017;67(2):263–271. doi: 10.1016/j.jhep.2017.03.039 28412293 10.1016/j.jhep.2017.03.039

[29] Jacobson IM, Lawitz E, Gane EJ, Willems BE, Ruane PJ, Nahass RG, et al Efficacy of 8 Weeks of Sofosbuvir, Velpatasvir, and Voxilaprevir in Patients With Chronic HCV Infection: 2 Phase 3 Randomized Trials. Gastroenterology. 2017;153(1):113–22. doi: 10.1053/j.gastro.2017.03.047 28390869 10.1053/j.gastro.2017.03.047

[30] Schnell G, Tripathi R, Beyer J, Reisch T, Krishnan P, Lu L, et al Hepatitis C Virus Genotype 4 Resistance and Subtype Demographic Characterization of Patients Treated with Ombitasvir plus Paritaprevir/Ritonavir. Antimicrob Agents Chemother. 2015;59(11):6807–6815. doi: 10.1128/AAC.01229-15 26282418 10.1128/AAC.01229-15 PMC4604390

[31] Halfon P, Mohamed S, Penaranda G, Khiri H, Chiche L, Nicolas C, et al Hepatitis C genotype 4R resistance-associated polymorphisms: The achilles heel of the nonstructural 5A inhibitors?: Correspondence. Hepatology. 2016;64(2):697–698. doi: 10.1002/hep.28611 27118238 10.1002/hep.28611

[32] AASLD. Recommendations for Testing, Managing, and Treating Hepatitis C. Aasld. 2016;1–51. http://hcvguidelines.org/sites/default/files/HCV-Guidance_July_2016_b.pdf

