# Supplementary material for: Evaluation of the cobas® GT hepatitis C virus genotyping assay in G1-6 viruses including low viral loads and LiPA failures
Source: PLoS One. 2018 Mar 22;13(3):e0194396. doi: 10.1371/journal.pone.0194396 (PMC5864039; doi:10.1371/journal.pone.0194396)
Supplement: S1 Table — This file includes sequencing results, results obtained with Roche assay on first and second pass and viral load for each sample. (DOCX) [file pone.0194396.s001.docx]

S1 table :

| Number | **Genotype** | **Result 1st pass** | **Result 2ond pass** | **VL** | **LOG VL** |
| --- | --- | --- | --- | --- | --- |
| RS1 | 3a | Indeterminate |  | 3715352 | 6,57 |
| RS2 | 1b | 1b |  | 7943282 | 6,9 |
| RS3 | 1b | 1b |  | 1065000 | 6,03 |
| RS4 | 1b | 1b |  | 1722000 | 6,24 |
| RS5 | 1a | 1a |  | NA | NA |
| RS6 | 1a | 1a |  | 4621 | 3,66 |
| RS7 | 3a | Indeterminate |  | 102100 | 5,01 |
| RS8 | 1b | 1b |  | 1071000 | 6,03 |
| RS9 | 1b | 1b |  | 535300 | 5,73 |
| RS10 | 4a | 4 |  | NA | NA |
| RS11 | 4d | 4 |  | 135000 | 5,13 |
| RS12 | 1a | 1a |  | 9525000 | 6,98 |
| RS13 | 3a | 3 |  | 9753000 | 6,99 |
| RS14 | 1a | 1a |  | NA | NA |
| RS15 | 1b | 1b |  | NA | NA |
| RS16 | 1a | Failed | 1a | 10400000 | 7,02 |
| RS17 | 4a | 4 |  | 1839000 | 6,26 |
| RS18 | 1a | 1a |  | 4916000 | 6,69 |
| RS19 | 4d | 4 |  | 285300 | 5,46 |
| RS20 | 1a | 1a |  | 1700000 | 6,23 |
| RS21 | 4a | 4 |  | 2990000 | 6,48 |
| RS22 | 1b | 1b |  | 1119000 | 6,05 |
| RS23 | 4r | 4 |  | 1818000 | 6,26 |
| RS24 | 1a | 1a |  | 1527000 | 6,18 |
| RS25 | 1b | 1b |  | NA | NA |
| RS26 | 1b | 1b |  | 146400 | 5,17 |
| RS27 | 1a | 1a |  | 16404 | 4,21 |
| RS28 | 1b | 1b |  | NA | NA |
| RS29 | 4d | 4 |  | 1082000 | 6,03 |
| RS30 | 1a | 1a |  | NA | NA |
| RS31 | 1a | 1a |  | NA | NA |
| RS32 | 3a | 3 |  | 1373000 | 6,14 |
| RS33 | 1b | 1b |  | 77541 | 4,89 |
| RS34 | 1a | 1a |  | 3626000 | 6,56 |
| RS35 | 1a | 1a |  | 67806 | 4,83 |
| RS36 | 3a | 3 |  | 1033000 | 6,01 |
| RS37 | 1b | 1b |  | 6201000 | 6,79 |
| RS38 | 1b | 1b |  | NA | NA |
| RS39 | 1a | 1a |  | 4576000 | 6,66 |
| RS40 | 4k | 4 |  | NA | NA |
| RS41 | 4d | Indeterminate |  | NA | NA |
| RS42 | 4a | 4 |  | 1055000 | 6,02 |
| RS43 | 1b | 1b |  | 2155000 | 6,33 |
| RS44 | 1a | 1a |  | 3698000 | 6,57 |
| RS45 | 4d | 4 |  | 445400 | 5,65 |
| RS46 | 2c | 2 |  | 49588 | 4,7 |
| RS47 | 1b | 1b |  | 367400 | 5,57 |
| RS48 | 1a | 1a |  | 2167000 | 6,34 |
| RS49 | 4d | Failed |  | 10121 | 4,01 |
| RS50 | 2c | 2 |  | 2990000 | 6,48 |
| RS51 | 1a | 1a |  | NA | NA |
| RS52 | 1a | 1a |  | 960200 | 5,98 |
| RS53 | 1b | 1b |  | 433200 | 5,64 |
| RS54 | 3a | 3 |  | 499000 | 5,7 |
| RS55 | 1a | 1a |  | 195300 | 5,29 |
| RS56 | 3a | 3 |  | 791200 | 5,9 |
| RS57 | 1a | 1a |  | 3792000 | 6,58 |
| RS58 | 1a | 1a |  | 9874000 | 6,99 |
| RS59 | 1a | 1a |  | 3939000 | 6,6 |
| RS60 | 1a | 1a |  | 8188000 | 6,91 |
| RS61 | 3a | 3 |  | 1811000 | 6,26 |
| RS62 | 1b | 1b |  | 5730000 | 6,76 |
| RS63 | 4d | 4 |  | 1894000 | 6,28 |
| RS64 | 1a | 1a |  | 1069000 | 6,03 |
| RS65 | 1b | 1b |  | 1213000 | 6,08 |
| RS66 | 1a | 1a |  | 542200 | 5,73 |
| RS67 | 1a | Failed | 1a | 1329000 | 6,12 |
| RS68 | 1a | Failed | 1a | 383100 | 5,58 |
| RS69 | 3a | Failed | 3 | 12381 | 4,09 |
| RS70 | 1a | 1a |  | 215500 | 5,33 |
| RS71 | 1a | Failed | Failed | 428900 | 5,63 |
| RS72 | 1b | 1b |  | 5273000 | 6,72 |
| RS73 | 1b | 1b |  | 336300 | 5,53 |
| RS74 | 1b | 1b |  | 990800 | 6 |
| RS75 | 1b | 1b |  | 9214000 | 6,96 |
| RS76 | 1b | 1b |  | 1291000 | 6,11 |
| RS77 | 3a | 3 |  | 1176000 | 6,07 |
| RS78 | 1b | 1b |  | 12580000 | 7,1 |
| RS79 | 1b | 1b |  | 1707000 | 6,23 |
| RS80 | 1a | 1a |  | 1809000 | 6,26 |
| RS81 | 1b | Failed | 1b | 7958000 | 6,9 |
| RS82 | 4a | 4 |  | 4400000 | 6,64 |
| RS83 | 3a | 3 |  | 62782 | 4,8 |
| RS84 | 1a | 1a |  | 1827000 | 6,26 |
| RS85 | 4d | 4 |  | 83177 | 4,92 |
| RS86 | 1a | 1a |  | 1264000 | 6,1 |
| RS87 | 1a | 1a |  | 1267000 | 6,1 |
| RS88 | 1b | 1b |  | 7960000 | 6,9 |
| RS89 | 4n | 4 |  | 1551000 | 6,19 |
| RS90 | 1b | 1b |  | 8296000 | 6,92 |
| RS91 | 4d | 4 |  | 552600 | 5,74 |
| RS92 | 4d | 4 |  | 1082000 | 6,03 |
| RS93 | 1a | 1a |  | 254700 | 5,41 |
| RS94 | 4a | Failed | Failed | 1235000 | 6,09 |
| RS95 | 1b | 1b |  | 180700 | 5,26 |
| RS96 | 1a | 1a |  | 2848000 | 6,45 |
| RS97 | 4d | Failed | Failed | 320400 | 5,51 |
| RS98 | 4d | 4 |  | 71036 | 4,85 |
| RS99 | 4a | 4 |  | 1220000 | 6,09 |
| RS100 | 6e | Indeterminate |  | 7881000 | 6,9 |
| RS101 | 1b | 1b |  | 5347000 | 6,73 |
