# Supplementary material for: Evaluation of the cobas® GT hepatitis C virus genotyping assay in G1-6 viruses including low viral loads and LiPA failures
Source: PLoS One. 2018 Mar 22;13(3):e0194396. doi: 10.1371/journal.pone.0194396 (PMC5864039; doi:10.1371/journal.pone.0194396)
Supplement: S2 Table — This file includes sequencing results, results obtained with Roche assay on first and second pass and viral load for each sample. (DOCX) [file pone.0194396.s002.docx]

S2 table :

| Number | **Genotype** | **Result 1st pass** | **Result 2ond pass** | **VL** | **LOG VL** |
| --- | --- | --- | --- | --- | --- |
| LF1 | 2i | 2 |  | 1394000 | 6,14 |
| LF2 | 4q | 4 |  | 101900 | 5,01 |
| LF3 | 4k | Invalid | Invalid | 1044000 | 6,02 |
| LF4 | 5a | 5 |  | 3710000 | 6,57 |
| LF5 | 2i | 2 |  | 1087000 | 6,04 |
| LF6 | 4o | Indeterminate |  | NA | NA |
| LF7 | 1g | Indeterminate |  | NA | NA |
| LF8 | 2i | 2 |  | NA | NA |
| LF9 | 1g | Indeterminate |  | NA | NA |
| LF10 | 2i | 2 |  | 308000 | 5,49 |
| LF11 | 4n | 4 |  | NA | NA |
| LF12 | 4b | 4 |  | NA | NA |
| LF13 | 5a | 5 |  | NA | NA |
| LF14 | 2i | 2 |  | NA | NA |
| LF15 | 2i | 2 |  | 3435000 | 6,54 |
| LF16 | 4r | Failed | 4 | NA | NA |
| LF17 | 2c | Failed | 2 | NA | NA |
| LF18 | 5a | 5 |  | NA | NA |
| LF19 | 4f | Failed | Failed | NA | NA |
| LF20 | 3h | Indeterminate |  | NA | NA |
| LF21 | 1g | Indeterminate |  | NA | NA |
| LF22 | 4f | 4 |  | NA | NA |
| LF23 | 2i | 2 |  | NA | NA |
| LF24 | 1d | 1b |  | NA | NA |
| LF25 | 2k | 2 |  | NA | NA |
| LF26 | 4f | 4 |  | NA | NA |
| LF27 | 4f | 4 |  | NA | NA |
| LF28 | 2b | 2 |  | NA | NA |
| LF29 | 1d | 1b |  | NA | NA |
| LF30 | 1i | Indeterminate |  | NA | NA |
| LF31 | 2i | 2 |  | NA | NA |
| LF32 | 4f | Failed |  | NA | NA |
| LF33 | 2i | 2 |  | NA | NA |
| LF34 | 3h | Indeterminate |  | NA | NA |
| LF35 | 4f | 4 |  | NA | NA |
| LF36 | 4f | 4 |  | NA | NA |
| LF37 | 4r | 4 |  | NA | NA |
| LF38 | 4b | Failed |  | NA | NA |
| LF39 | 2k/1b | 2k/1b |  | NA | NA |
