# Supplementary material for: Evaluation of the cobas® GT hepatitis C virus genotyping assay in G1-6 viruses including low viral loads and LiPA failures
Source: PLoS One. 2018 Mar 22;13(3):e0194396. doi: 10.1371/journal.pone.0194396 (PMC5864039; doi:10.1371/journal.pone.0194396)
Supplement: S3 Table — This file includes sequencing results, results obtained with Roche assay on first and second pass and viral load for each sample. (DOCX) [file pone.0194396.s003.docx]

S3 table :

| Number | **Genotype** | **Result 1st pass** | **Result 2ond pass** | **VL** | **LOG VL** |
| --- | --- | --- | --- | --- | --- |
| LVL1 | 1b | 1b |  | 39 | 1,59 |
| LVL2 | 1b | 1 |  | 17 | 1,23 |
| LVL3 | 3a | 3 |  | 66 | 1,82 |
| LVL4 | 1b | 1b |  | 114 | 2,06 |
| LVL5 | 1b | 1 |  | 43 | 1,63 |
| LVL6 | 1a | Invalid | Failed | 52 | 1,72 |
| LVL7 | 3a | 3 |  | 132 | 2,12 |
| LVL8 | 1a | 1 |  | 396 | 2,6 |
| LVL9 | 4g | Invalid |  | 25 | 1,4 |
| LVL10 | 4d | Invalid |  | 91 | 1,96 |
| LVL11 | 4a | 4 |  | 898 | 2,95 |
| LVL12 | 1b | 1b |  | 140 | 2,15 |
| LVL13 | 1a | Indeterminate |  | 41 | 1,61 |
| LVL14 | 4a | Invalid | Failed | 145 | 2,16 |
| LVL15 | 1b | 1 |  | 69 | 1,84 |
| LVL16 | 1a | 1a |  | 69 | 1,84 |
| LVL17 | 1a | 1a |  | 160 | 2,2 |
| LVL18 | 1b | Invalid |  | 72 | 1,86 |
| LVL19 | 1a | 1a |  | 687 | 2,84 |
| LVL20 | 3a | 3 |  | 364 | 2,56 |
| LVL21 | 3a | Failed | Failed | 29 | 1,46 |
| LVL22 | 1a | Indeterminate |  | 59 | 1,77 |
| LVL23 | 4a | 4 |  | 111 | 2,05 |
| LVL24 | 1a | Invalid |  | 26 | 1,41 |
| LVL25 | 4d | Indeterminate |  | 31 | 1,49 |
| LVL26 | 1a | 1a |  | 171 | 2,23 |
| LVL27 | 3a | Invalid |  | 22 | 1,34 |
| LVL28 | 3a | Invalid |  | 46 | 1,66 |
| LVL29 | 1b | Invalid |  | 174 | 2,24 |
| LVL30 | 1b | 1b |  | 241 | 2,38 |
| LVL31 | 1a | 1 |  | 344 | 2,54 |
| LVL32 | 1a | Invalid |  | 55 | 1,74 |
| LVL33 | 1b | 1b |  | 143 | 2,16 |
| LVL34 | 1b | 1b |  | 69 | 1,84 |
| LVL35 | 1a | Indeterminate |  | 261 | 2,42 |
| LVL36 | 1a | Indeterminate |  | 34 | 1,53 |
| LVL37 | 1b | Invalid |  | 27 | 1,43 |
| LVL38 | 1b | 1b |  | 362 | 2,56 |
| LVL39 | 1b | 1b |  | 60 | 1,78 |
| LVL40 | 1b | Invalid |  | 26 | 1,41 |
| LVL41 | 1e | Invalid |  | 20 | 1,3 |
| LVL42 | 1b | Invalid |  | 51 | 1,71 |
